# *N* ^6^‐methyladenosine‐modified circFUT8 competitively interacts with YTHDF2 and miR‐186‐5p to stabilize FUT8 mRNA to promote malignant progression in lung adenocarcinoma

**DOI:** 10.1111/1759-7714.15086

**Published:** 2023-09-05

**Authors:** Gaochao Dong, Yingkuan Liang, Bing Chen, Te Zhang, Hui Wang, Yuzhong Chen, Yijian Zhang, Feng Jiang, Yaping Wang

**Affiliations:** ^1^ Department of Medical Genetics, Medical School Nanjing University Nanjing China; ^2^ Department of Thoracic Surgery Nanjing Medical University Affiliated Cancer Hospital & Jiangsu Cancer Hospital & Jiangsu Institute of Cancer Research Nanjing China; ^3^ Jiangsu Key Laboratory of Molecular and Translational Cancer Research Cancer Institute of Jiangsu Province Nanjing China; ^4^ Department of Thoracic Surgery The First Affiliated Hospital of Soochow University Suzhou China; ^5^ The Fourth Clinical College of Nanjing Medical University Nanjing China; ^6^ Jiangsu Key Laboratory of Molecular Medicine, Medical School Nanjing University Nanjing China

**Keywords:** circFUT8, lung adenocarcinoma, miR‐186‐5p, *N* ^6^‐methyladenosine, YTHDF2

## Abstract

**Background:**

Lung cancer is the leading cause of cancer related to mortality worldwide, and the main pathological type is lung adenocarcinoma (LUAD). Circular RNAs (circRNAs) have been reported to be modified by *N* ^6^‐methyladenosine (m6A), which is involved in the progression of diverse tumors. However, the crosstalk between circRNAs and m6A modification has not been well elucidated in LUAD.

**Methods:**

MeRIP‐seq and YTHDF2‐RIP‐seq datasets were explored to identify candidate circRNAs modified by YTHDF2. Dual‐luciferase reporter assay, RIP, and rescue assays were performed to explore the relationship between circFUT8 and its parent mRNA of FUT8. In vitro and in vivo experiments were utilized to uncover the function of circFUT8.

**Results:**

In this study, we identified a novel m6A‐modified circFUT8, derived from exon 3 of FUT8, which was elevated in tumor tissues compared with adjacent noncancerous tissues. The m6A reader YTHDF2 recognized and destabilized circFUT8 in an m6A‐dependent manner. YTHDF2 also combined with the line form of FUT8 (mFUT8), and circFUT8 competitively interacted with YTHDF2, blunting its binding to mFUT8, to stabilize the mRNA level of FUT8. Additionally, circFUT8 sponged miR‐186‐5p to elevate the expression of mFUT8. Finally, we revealed that circFUT8 promoted the malignant progression of LUAD dependent on the oncogenic function of FUT8.

**Conclusions:**

These findings identified a novel m6A‐modified circFUT8 recognized and destabilized by YTHDF2, which competitively interacted with YTHDF2 and miR‐186‐5p to stabilize FUT8 mRNA to promote malignant progression in LUAD.

## Graphical abstract

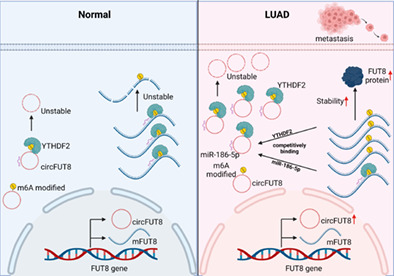

## INTRODUCTION

Lung cancer remains the most common cause of cancer related to mortality worldwide, which is divided into small cell lung cancer and non‐small cell lung cancer (NSCLC) based on pathology.^1^ Due to the development of targeted and immunotherapy, the treatment paradigm for patients with lung adenocarcinoma (LUAD), the most prevalent subtype of lung cancer, has been dramatically altered.^2^, ^3^ However, owing to the perplexing pathogenesis and heterogeneity of LUAD, the 5‐year survival rate remains unsatisfactory.^4^ A proposal for a novel approach to explore the molecular mechanism is therefore urgently required.

With the development of high‐throughput sequencing technology and novel bioinformatic algorithms, circRNAs, covalently closed RNA molecules, have been detected in a variety of tissues, including brain, lung, liver, and so on.^5^, ^6^, ^7^ Because of the growing understanding of its characterization, the highly stable circRNAs have attracted a great deal of attention of researchers in the field of cancer research.^8^ Chen and colleagues identified that circUSP7 secreted by NSCLC cells in an exosomal manner induced CD8+ T cell dysfunction via sponging miR‐934 to upregulate the expression of SH2‐containing protein tyrosine phosphatase 2 (SHP2).^9^ Our group also revealed that circDCUND4, downregulated in LUAD samples under the mediation of DExH‐box helicase 9 (DHX9), facilitated the interaction of HuR protein and thioredoxin‐interaction protein (TXNIP) mRNA to suppress the metastasis of LUAD.^10^ The crosstalk between *N* ^6^‐methladenosime (m6A) and circRNAs has been noticed to influence many aspects of circRNAs including biogenesis, localization, and translation.^11^, ^12^, ^13^ However, circRNAs modified by m6A have not been well elucidated in LUAD.

RNA m6A methylation, which has been widely studied as vital post‐transcriptional epigenetic modulators of gene expression in eukaryotes, is a dynamic progress accomplished by the m6A methyltransferase complex, referred to “writers,” including METTL3, METTL14, and WTAP and so on, and the m6A demethylases, referred to “erasers,” composed of FTO and ALKBH5.^14^, ^15^ It also can be recognized by “readers,” which is a field of extreme interest due to the fate determination of target RNAs.^16^ YTH domain‐containing protein 1 (YTHDC1), a conserved m6A reader protein, has been reported to promote the transportation of circNSUN2 from the nucleus to the cytoplasm in a m6A methylation‐dependent manner.^17^ YT521‐B homology (YTH) domain family protein 3 (YTHDF3) together with initiation factor eIF4G2 to promote efficient initiation of protein translation from circRNAs, which was enhanced by METTL3/14 and inhibited by FTO.^18^ Little is known about the biological function of circRNAs recognized by the m6A protein YTHDF2 in LUAD.

In this study, we screened the MeRIP‐seq, YTHDF2‐RIP‐seq, and identified that the upregulated circFUT8 in LUAD tumor samples, derived from the exon 3 of FUT8, could be recognized by m6A reader protein YTHDF2. We also revealed that circFUT8 increased the stability of the line form, mFUT8, in a m6A dependent manner. Additionally, circFUT8 sponged miR‐186‐5p to elevate the expression of mFUT8. Moreover, circFUT8 promoted the malignant progression of LUAD dependent on the oncogenic function of FUT8. Our study provides a novel insight into the regulatory network between the circRNAs and their host genes in post‐transcription.

## METHODS

### Tissue collection

The Jiangsu Cancer Hospital's Ethics Committee approved the experiment, which adhered to the necessary standards. Between 2017 and 2021, 40 paired human lung adenocarcinoma (LUAD) and adjacent tissues (ANT) were obtained from the Affiliated Cancer Hospital of Nanjing Medical University (Nanjing, China). With the help of experienced pathologists, tissues were established as reliable diagnoses. The procedures for the collection and use of tissues were performed in accordance with the guidelines of the Declaration of Helsinki, 2013. All samples were obtained from biobank of Jiangsu Cancer Hospital (Jiangsu Institute of Cancer Research and The Affiliated Cancer Hospital of Nanjing Medical University). All patients had signed informed consent for donating their samples.

### Cell cultures

The human bronchial epithelial cell (HBE cells, cat. ZQ0001) and LUAD cell lines including A549 (cat. SCSP‐503), PC9 (cat. SCSP‐5085), H1299 (cat. SCSP‐589), SPC‐A1 (cat. ZQ0018), and H1975 cells (cat. SCSP‐597) were taken from the Chinese Academy of Sciences Cell Bank and authenticated using DNA fingerprinting, as well as being mycoplasma‐free. HBE, A549, H1299, and H1975 cells were cultured in RPMI‐1640 medium (Keygen Biotech) supplemented with 10% fetal bovine serum (FBS: Gibco). PC9 and SPC‐A1 were cultured with Dulbecco's modified Eagle medium (DMEM: Keygen Biotech) supplemented with 10% FBS. In a humidified 37°C incubator, cells were stored in a 5% CO_2_ environment. Guangzhou Biotech Corporation authenticated no mycoplasma was found in the cells.

### CircRNAs identified

We identified the circRNAs from MeRIP‐seq (GSE85324),^19^ YTHDF2 RIP‐seq (GSE49339).^20^ The differential expression circRNAs in LUAD data were obtained from a circRNAs microarray including five LUAD tissues and adjacent tissues (GSE85324).

### Quantitative real‐time polymerase chain reaction and RNase R treatment

TRIzol reagent was used to extract RNA from cells (Fisher Scientific). A cell fragmentation isolation kit was used to extract nuclear and cytoplasmic RNA and protein (Fisher Scientific). After incubating 1 μg of total RNA at 37°C, the RNase R solution was added, the mixture was incubated in 70°C 30 min, and the reaction was allowed to continue (Geneseed). Then, qRT‐PCR was carried out using SYBR green master mix with random hexamers for reverse transcription (Takara) or oligo(dT)18 primers (Qiagen: Takara). The amplification and analysis were carried out using the step one Plus Real‐Time PCR technique (ABI) and the corresponding instruments. 2^−ΔΔCT^ was used to measure the expression. The PCR products were purified using a PCR purification kit (Qiagen). Table S1 contains a list of primer sequences.

### Actinomycin D assay

A549 cells were planted in five wells in 24‐well plates (5 × 104 cells per well). Actinomycin D was applied to cells after 24 h (2 μg/mL, Abcam) for 0, 4, 8, 12, and 24 h, respectively. After that, the relative RNA levels of circFUT8 and mFUT8 were analyzed by qRT‐PCR and normalized to the values of the 0 h group.

### Overexpression or knockdown of genes

Human circFUT8 linear sequence was obtained from LUAD tissues by PCR and inserted into plasmid vector pcDNA 3.1 (Hanbioa). The small interfering RNA (siRNA) of circFUT8, mFUT8 were provided by RiboBio. The target sequences are supplied in Table S1. According to the target sequence of si‐circFUT8, cloned a short hairpin RNA (shRNA) (sh‐circFUT8) into pGFP‐u6 vector. A Lipofectamine 3000 kit (Invitrogen) was used to perform transient transfection of the shRNA or overexpressing plasmids, and the Lipofectamine iMax kit (Invitrogen) was used to perform transient transfection of siRNA.

### Western blotting

To summarize, total protein was purified from cells using a radioimmunoprecipitation assay (RIPA: Thermo Fisher Scientific) with a cocktail of proteinase and phosphatase inhibitors (Thermo Fisher Scientific) according to the manufacturer's protocol. SDS‐PAGE gels were used to separate equal quantities of protein lysates, which were then transferred to a polyvinylidene fluoride (PVDF) membrane (Millipore). After incubating with a primary antibody overnight at 4°C, the membranes were hybridized with a secondary antibody for 1 h at room temperature. The LI‐COR Odyssey CLx imaging system produces a signal number for each band identified on a western blot generated by the near‐infrared fluorescent detection of secondary antibodies used. The antibody information is listed in Table S2.

### RNA immunoprecipitation assay (RIP)

According to the manufacturer's instructions, the Magna RIPTM RNA‐binding protein immunoprecipitation kit was used in the RIP assay (Millipore). In summary, 2 × 10^7^ cells were treated in lysis buffer on ice for 10 min. At room temperature, magnetic beads were incubated with 5 μg antibody, and then cultured with the cell lysates overnight at 4°C. The immunoprecipitated RNAs were collected after being treated with proteinase K. QRT‐PCR was used to evaluate the abundance of circFUT8. For RIP, m6A and YTHDF2 antibodies were used. The coprecipitated RNA was monitored using qRT‐PCR. Table S2 contains information on antibodies.

### RNA pulldown

The biotin‐labeled RNA probes of circFUT8 and scramble were synthesized by GenePharma Company. In the RNA pulldown assay, a biotinylated pulldown kit was used (Thermo Fisher Scientific). In brief, 2 × 10^7^ cells were incubated in lysis solution on ice for 30 min. For 30 min, the streptavidin‐coated magnetic beads were incubated with biotinylated probes at room temperature. After putting the beads‐probe complex to the lysis, it was mixed at 4°C for 2 h. The sealed beads' attached proteins were eluted. RiboBio provided the biotin‐modified miRNA probe. The sequences of probes are provided in Table S1.

### RNA‐fluorescence in situ hybridization assay

RNA‐fluorescence in situ hybridization (FISH) assays were carried out according to the manufacturer's instructions using an RNA‐FISH kit (GenePharma). GenePharma created a Cy3‐labeled antisense probe against the back‐splicing junction site of circFUT8 and Cy3‐labeled probe of FUT8 liner transcript. In brief, A549 cells were incubated in 4% paraformaldehyde. Cells were blocked and hybridized in hybridization buffer with Cy3‐labeled probe at 37°C overnight after prehybridization with 0.5% Triton X‐100. Cells were stained with 4′, 6‐diamidino‐2‐phenylindole (DAPI: 300 nmol/L). The probes are provided in Table S1.

### Real‐time cell analysis (RTCA)

For migration assay, the “xCELLigence” system (Roche Applied Sciences and ACEA Biosciences) including 16‐well CIM‐16 (Roche Diagnostics GmbH) was used. Initially, the CIM‐16 plate was locked at 37°C after 165 μL medium and 30 μL of serum‐free media and 5% CO_2_ for 30 min to obtain equilibrium, respectively, then were introduced to the lower and upper chambers. Then, 4 × 10^4^ cells were resuspended in serum‐free 100 μL medium to seed in the upper chamber.

### Transwell and Matrigel assay

Cells (4 × 10^4^) were sown in the upper transwell assay chambers of millipore 8 μm pore filters (for the migration assay). For the invasion assay, 4 × 10^4^ cells were plated in serum‐free media into the upper matrigel assay chambers with a membrane coated with Matrigel (Corning). Medium containing 10% FBS was present in the lower chamber. Nonmigrating or noninvading cells were removed carefully after culturing at 37°C for 24 h for migration and 48 h for invasion. Cells that migrated to the bottom of the membrane were then fixed with 4% paraformaldehyde, stained with crystal violet solution for 30 min, and visualized under a microscope at ×100 magnification. The experiments were conducted in triplicate.

### Wound‐healing assay

Transfected cells were cultured in six‐well plates. After the cells reached 90% confluence, a standard 200 μL pipette tip was subsequently utilized to scratch linear wounds. In addition, the cell monolayers were cultivated in FBS‐free medium. After scratching, the images of the wound closure were captured at 0, and 36 h. The experiments were conducted in triplicate.

### In vivo animal model and growth, metastasis assays

Thirty female BALB/c nude mice weighing 18–22 g were randomly assigned to six groups. PC9‐Mock, PC9‐circFUT8, A549‐sh‐scramble, and A549‐sh‐circFUT8 cells were prepared as a suspension of 4 × 10^5^ cells in 200 μL saline, respectively, and injected into the tail vein. Mice were sacrificed at 6 weeks post injection and examined microscopically by hematoxylin and eosin (H&E) staining for the development of lung metastases. The animal study was carried out according to the State Food and Drug Administration of China regulations on animal care (IACUC‐2012002‐1). Animals were sorted only by treatment, and no exclusion or inclusion of an animal was predetermined.

### Statistical analysis

All statistical analyses used SPSS 25.0. Data are represented as mean ± SD. An unpaired, two‐tailed student's *t*‐test was used for comparison between the two groups. Two‐way ANOVA with Tukey test was performed to compare multiple groups. Differences with *p* < 0.05 were considered statistically significant.

## RESULTS

### Screening and characteristics of circFUT8 in LUAD

**FIGURE 1:**
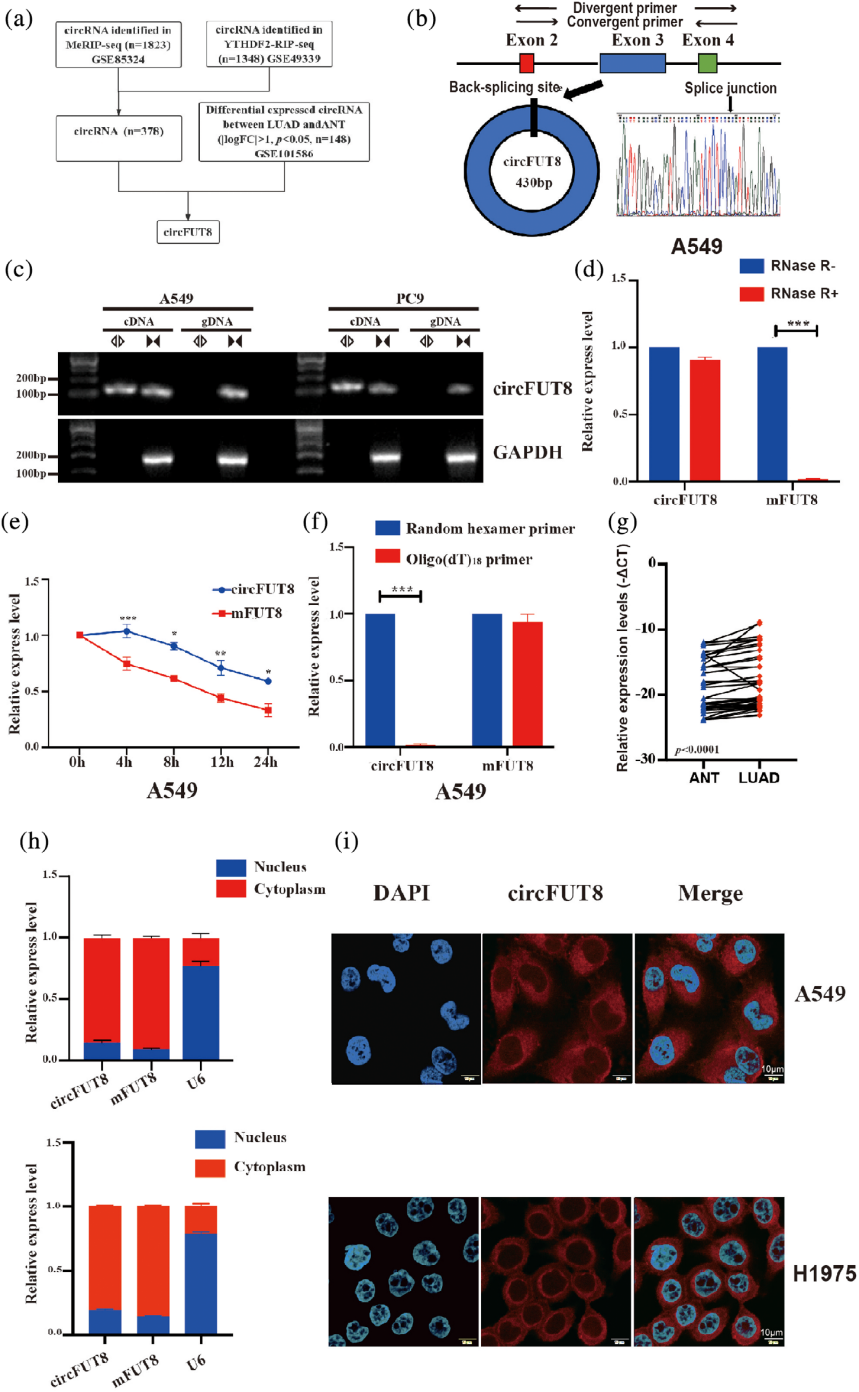
Screening and characteristics of circFUT8 in lung adenocarcinoma (LUAD). (a) The flow diagram demonstrated the screen process of circFUT8. (b) Scheme illustrating the production of circFUT8. CircFUT8 is formed by back splicing from exon 3 of the FUT8 gene. The expression of circFUT8 is validated by sanger sequencing, the arrow indicates the junction site of circFUT8. (c) Quantitative real‐time polymerase chain reaction (qRT‐PCR) assay indicating the detection of circFUT8 using divergent and convergent primers from cDNA or genomic DNA (gDNA) of cancer cell lines A549. (d) PCR analysis for the expression of circFUT8 and mFUT8 after treatment with RNase R in the total RNA of A549 cells. (e) The relative RNA levels of circFUT8 and mFUT8 were analyzed by qRT‐PCR after treatment with actinomycin D at the indicated time points in A549 cells. (f) Random hexamer or oligo(dT)18 primer used in reverse transcription experiments, and the analysis of the circFUT8 and mFUT8 levels by qRT‐PCR. (g) The expression levels of circFUT8 in 40 paired LUAD and ANT using qRT‐PCR. (h, i) The expression levels of circFUT8 in the nuclear and cytoplasmic fraction of A549 and H1975 cells using qRT‐PCR and fluorescence in situ hybridization (FISH) (red). 4′, 6‐diamidino‐2‐phenylindole (DAPI) stained nuclei. Scale bar, 10 μm.

To identify the circRNAs modified by *N* ^6^‐methyladenosine, we integrated the publish data, which contains 1823 circRNAs with m6A modification (MeRIP‐seq, GSE85324), and the other contains 1348 circRNAs interacted with YTHDF2 (YTHDF2‐RIP‐seq, GSE49339), the “reader” protein of m6A. Overlapping these two series of circRNAs, we obtained 378 candidate circRNAs, which could be modified by m6A and recognized by m6A reader protein YTHDF2. In addition, we also screened the differentially expressed circRNAs in tumor and paratumoral tissues (GSE101586),^21^ and selected the circFUT8 (hsa_circ_0003028) for its significant differential expression (Figures 1a and S1A). CircFUT8 is derived from exon 3 of a protein‐coding gene FUT8, which was confirmed by Sanger sequencing (Figure 1b). The expression levels of circFUT8 in LUAD cell lines were detected, which revealed that circFUT8 was highly enriched in A549 cells and weakly enriched in PC9 cells (Figure S1B). The circular structure of circFUT8 was also identified through PCR analysis in LUAD cancer cell lines (Figure 1c). CircFUT8 was more resistant when treated with RNase R or the transcription inhibitor actinomycin D (Figures 1d, e and S1C, D), compared with the line form mFUT8 in A549 and H1975 cells. Additionally, circFUT8 expression was significantly decreased when detected by oligo‐dT primers compared with that by random primers, which indicated that circFUT8 did not have the poly‐A tail (Figures 1f and S1E). We also used divergent primers to detect the expression of circFUT8 in 40 paired LUAD and adjacent noncancerous tissues, which revealed that circFUT8 expression was elevated in tumor tissues (Figure 1g), consistent with the result of screening before. Nuclear/cytosolic fractionation assay was performed to reveal that the principal localization of circFUT8 was in the cytosol (Figure 1h), which was further confirmed by fluorescence in situ hybridization (FISH) assay (Figure 1i).

### Recognition of circFUT8 by YTHDF2 in an m6A‐dependent manner

**FIGURE 2:**
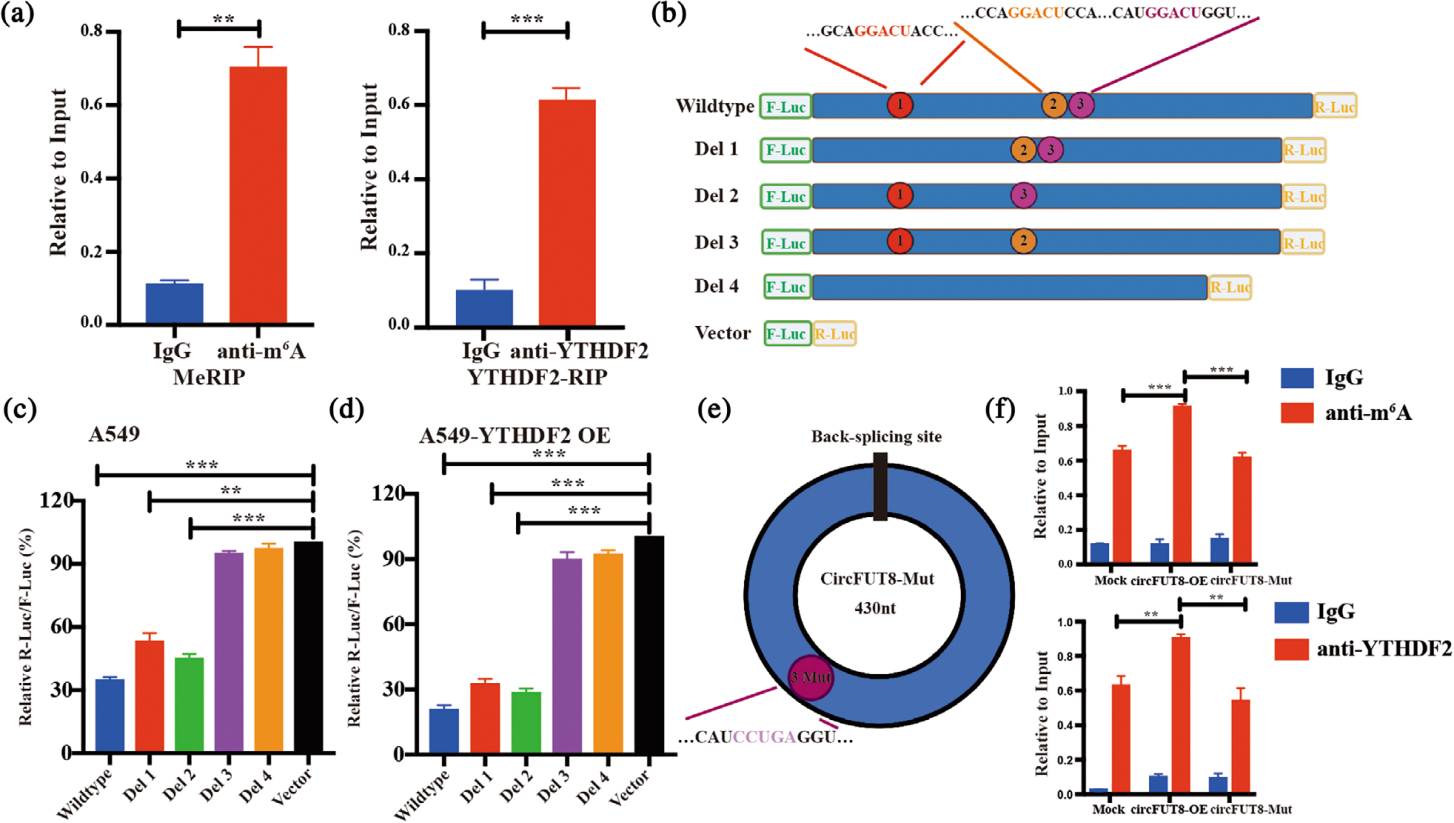
Validation of circFUT8 region modified by m6A. (a) Left, *N* ^*6*^‐methyladenosine RNA immunoprecipitation (MeRIP) assay shows the m6A modification of circFUT8. Right, the interaction between YTHDF2 and circFUT8 was validated by RIP assay. (b) Scheme illustrating the m6A binding site candidate and the deletion plasmid, respectively. (c, d) The dual‐luciferase reporter assay indicated that the del 3 site was the read site of YTHDF2 on circFUT8. (e) Scheme illustrating the mutation circFUT8 in del 3. (f) The MeRIP and anti‐YTHDF2 RIP assay indicated that mut circFUT8 could not be effectively enriched by anti‐m6A and anti‐YTHDF2.

As m6A enzymes commonly modify corresponding transcripts through directly binding to them,^22^ the MeRIP assay indicated that circFUT8 underwent m6A modification, and circFUT8 enrichment was also observed in the YTFDF2‐RIP assay, which revealed that the m6A reader YTHDF2 interacted with circFUT8 (Figure 2a). Using SRAMP software,^23^ we found three possible m6A sites (region 1, 2, and 3) containing GGACU sequences in the transcript of circFUT8 (Figure 2b). To validate the core region modified by m6A, we constructed the truncated plasmids with luciferase including del 1 (containing region 2 and 3), del 2 (containing region 1 and 3), del 3 (containing region 1 and 2), del 4 (no contain), and vector as positive control (Figure 2b). YTHDF2 was previously proved to induce the degradation of liner RNA,^24^, ^25^ thus, we performed a dual‐luciferase reporter assay to explore the m6A modified site in circFUT8. The dual‐luciferase reporter assay revealed that region 3, not region 1 or 2, was crucial for m6A modification (Figure 2c, d). To substantiate the interaction between YFHDF2 and circFUT8 dependent on m6A modification, the core sequence in region 3 was mutated, which decreased the circFUT8 enrichment in Me‐RIP or YTFDF2‐RIP assay (Figure 2e, f).

### YTHDF2 both interacts with circFUT8 and mFUT8 to mediate degradation

**FIGURE 3:**
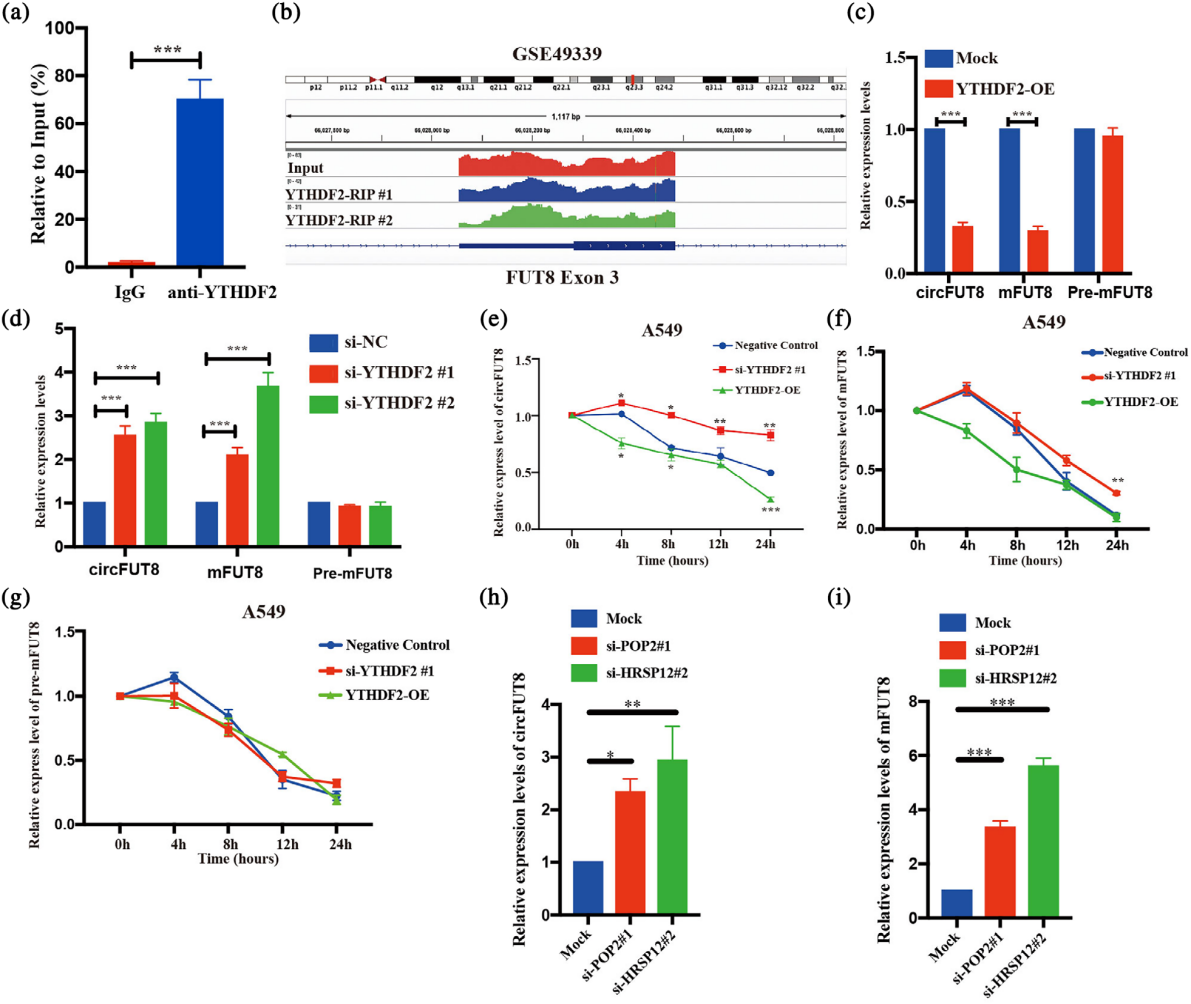
YTHDF2 interacts with both circFUT8 and mFUT8 to mediate degradation. (a) The RNA immunoprecipitation (RIP) assay demonstrated the interaction between YTHDF2 and mFUT8. (b) The RIP‐seq peaks were enriched in exon 3 of mFUT8. (c, d) The expression levels of YTHDF2 significantly suppressed the expression of circFUT8 and mFUT8. (e–g) The actinomycin D treatment in A549 revealed that YTHDF2 regulated both circFUT8 and mFUT in post‐transcription level. (h, i) The expression of POP1 and HRSP12 significantly suppressed the expression of circFUT8 and mFUT8.

Due to the sequence similarity between circFUT8 and the line transcript mFUT8, we also observed the mFUT8 enrichment in the YTHDF2‐RIP assay (Figure 3a, b). It has been reported that YTHDF2 facilitates the degradation of circRNAs.^26^ First, we assessed the transcriptional level of circFUT8 and mFUT8 when manipulating the expression of YTFDH2. The efficiency of YTHDF2 overexpression and knockdown in LUAD cells were verified (Figure S2A, B). It was observed that overexpression of YTHDF2 decreased the expression of circFUT8 and mFUT8, and the opposite result was confirmed with the knockdown of YTHDF2 (Figure 3c, d). We also observed that perturbation of YTHDF2 expression could not affect the expression of pre‐mFUT8, which implicated that YTHDF2 had no effect on the transcription of FUT8. Additionally, we identified that both circFUT8 and mFUT8 were destabilized by YTHDF2 overexpression, and silencing of YTHDF2 increased the stability of circFUT8 and mFUT8, not the pre‐mFUT8 (Figure 3e–g). These results confirmed that YTHDF2 could interact with both circFUT8 and mFUT8 to decrease their expression.

Because the degradation of circRNAs via YTHDF2 is dependent on HRSP12‐RNase P/MRP axis, we knocked down the expression of HRSP12 and POP2, the core protein of RNase P/MRP complexes, respectively (Figure S2C). We found that both RNA fragments of circFUT8 and mFUT8 were increased when suppressing the expression of HRSP12 or POP2 (Figures 3h, i and S2D, E).

### CircFUT8 interacts with YTHDF2 to upregulate expression of mFUT8

**FIGURE 4:**
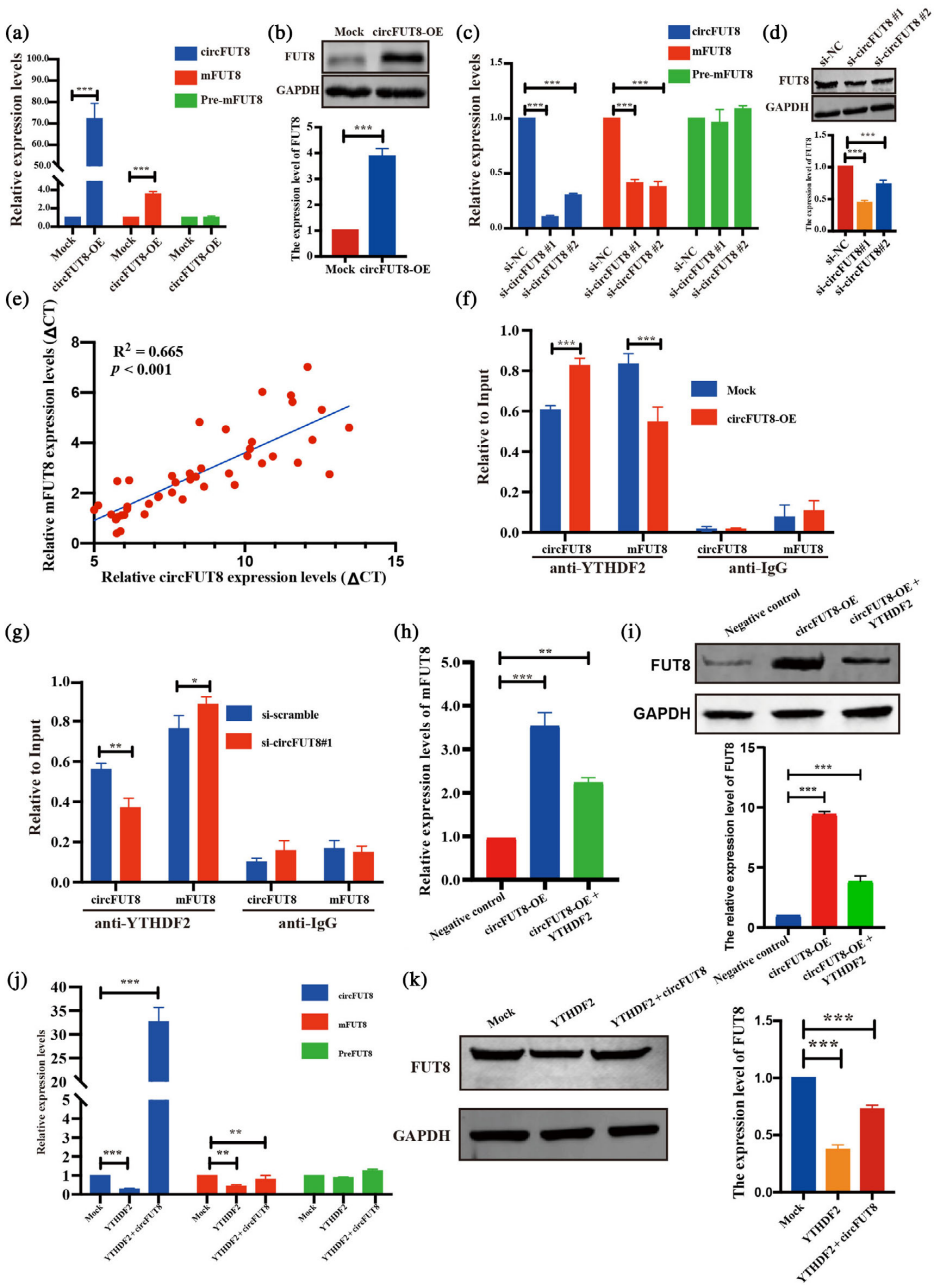
CircFUT8 sponges YTHDF2 to upregulate expression of mFUT8. (a, b) Quantitative real‐time polymerase chain reaction (qRT‐PCR) and western blot assays indicated that circFUT8 promoted the expression of mFUT8. (c, d) The expression of mFUT8 was significantly decreased upon circFUT8 knockdown. (e) The expression of mFUT8 in 40 paired lung adenocarcinoma (LUAD) tissues, showed a significant positive correlation to circFUT8. (f, g) CircFUT8 and mFUT8 competitively bind with YTHDF2. (h, i) The qRT‐PCR and western blot assays revealed that circFUT8 promoted the expression of mFUT8 ability in A549 cells and was partly blocked by YTHDF2 overexpression. (j, k) CircFUT8 could rescue the expression of mFUT8 by competitively binding with YTHDF2 in mRNA and protein levels.

It has been reported that circRNAs regulate the parental line transcript expression through a variety of pathways including interacting with RNA binding proteins and alternative splicing.^27^ In this study, we hypothesized that circFUT8 regulated expression of mFUT8 via m6A modification. First, we overexpressed the expression of circFUT8, which increased the expression of mFUT8 (Figure 4a, b), while we knocked down the expression of circFUT8, which suppressed the expression of mFUT8 (Figure 4c, d). A positive correlation was also observed between circFUT8 and mFUT8 in LUAD tumor tissues (Figure 4e). Moreover, overexpression of circFUT8 decreased the enrichment of YTHDF2 in mFUT8 and knockdown of circFUT8 increased the enrichment of YTHDF2 in mFUT8 (Figure 4f, g), which revealed that circFUT8 competitively binds YTHDF2 protein to regulate the stability of mFUT8. The increased expression level of FUT8, induced by circFUT8 overexpression, was rescued by YTHDF2 overexpression (Figure 4h, i). Additionally, the rescue experiments revealed that overexpression of circFUT8 partly neutralized the decreased expression of mFUT8 induced by YTHDF2 expression (Figure 4j, k). These results revealed that circFUT8 regulated the expression of mFUT8 via interacting with m6A reader protein YTHDF2.

### CircFUT8 sponges miR‐186‐5p to upregulate expression of mFUT8

**FIGURE 5:**
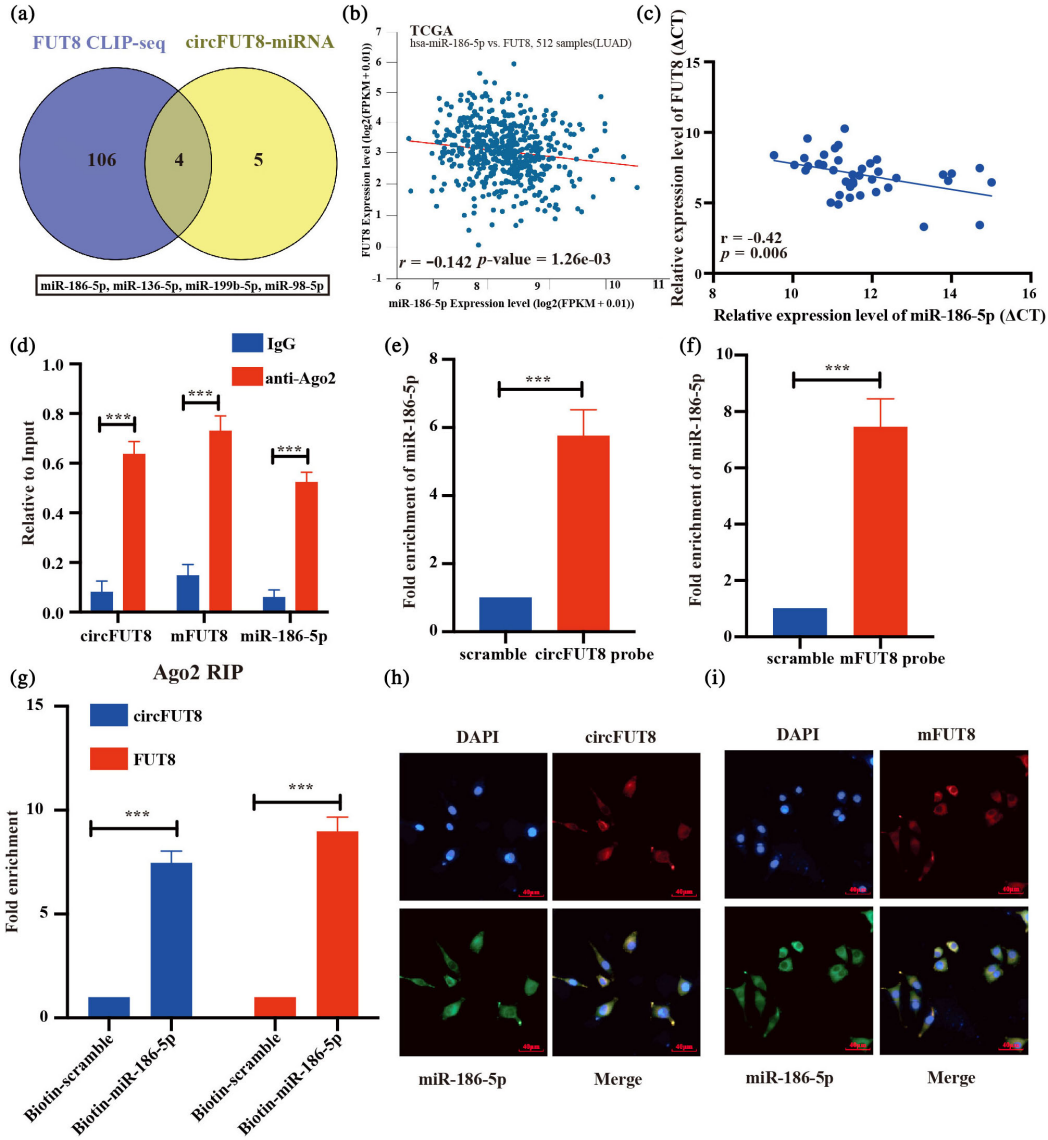
CircFUT8 sponges miR‐186‐5p to upregulate expression of mFUT8. (a) Venn diagram showing the common candidate miRNAs from both FUT8 CLIP‐seq and miRNA data, which was predicted. (b) The expression levels of miR‐186‐5p were significantly negatively correlated to mFUT8 in the TCGA database and our own cohort (c). (d) The RNA immunoprecipitation (RIP) assay demonstrated that Ago2 could efficiently gather the circFUT8, mFUT8 and miR‐186‐5p. (e, f) The pulldown assays verified the interaction between miR‐186‐5p and circFUT8, miR‐186‐5p, and mFUT8, respectively. (g) The biotin modified miR‐186‐5p could significantly bind to circFUT8 and mFUT8. (h) RNA‐fluorescence in situ hybridization (FISH) and immunofluorescence staining assay showing colocalization of circFUT8 (red) and miR‐186‐5p (green) in A549 cells, and 4′, 6‐diamidino‐2‐phenylindole (DAPI) stained nuclei (blue). Scale bar: 40 μm. (i) RNA‐FISH and immunofluorescence staining assay showing colocalization of mFUT8 (red) and miR‐186‐5p (green) in A549 cells, and DAPI stained nuclei (blue). Scale bar: 40 μm.

In the previous experiment, we observed that circFUT8 partly rescued the expression of mFUT8 via m6A modification. We proposed that circFUT8 also regulated mFUT8 expression through other avenues. The most proposed biological function of the circRNAs is that they act as miRNA sponges.^27^ First, we screened the CLIP‐seq data of mFUT8 and analyzed the potential miRNA with targeting sites for circFUT8 through bioinformatic analysis, and four miRNAs were selected as the candidate miRNAs including miR‐186‐5p, miR‐136‐5p, miR‐199b‐5p, and miR‐98‐5p, which synchronously interacted with circFUT8 and mFUT8 (Figure 5a). We also analyzed the correlation between mFUT8 and the four candidate miRNAs, and we selected miR‐186‐5p as the sponge miRNA for its significant negative correlation with mFUT8 both in TCGA‐LUAD and our LUAD tumor tissues (Figure 5b, c). AGO2 always mediated the circRNAs sponging miRNA and miRNA cleaving target transcripts.^28^ The RIP experiment revealed that circFUT8, mFUT8, and miR‐186‐5p were all significantly enriched under the action of anti‐AGO2 (Figure 5d). We also constructed the biotin‐probe labeled circFUT8 and mFUT8, respectively, and we performed pulldown assays to identify that both circFUT8 and mFUT8 could directly interact with miR‐186‐5p (Figure 5e, f). Additionally, the pulldown experiment revealed that the biotinylated miR‐186‐5p also directly interacted with circFUT8 and mFUT8, too (Figure 5g). Moreover, the RNA FISH assay revealed that circFUT8 and mFUT8 colocalized in the cytoplasm with miR‐186‐5p, respectively (Figure 5h, i). The above results indicate that circFUT8 and mFUT8 can both directly interact with miR‐186‐5p.

**FIGURE 6:**
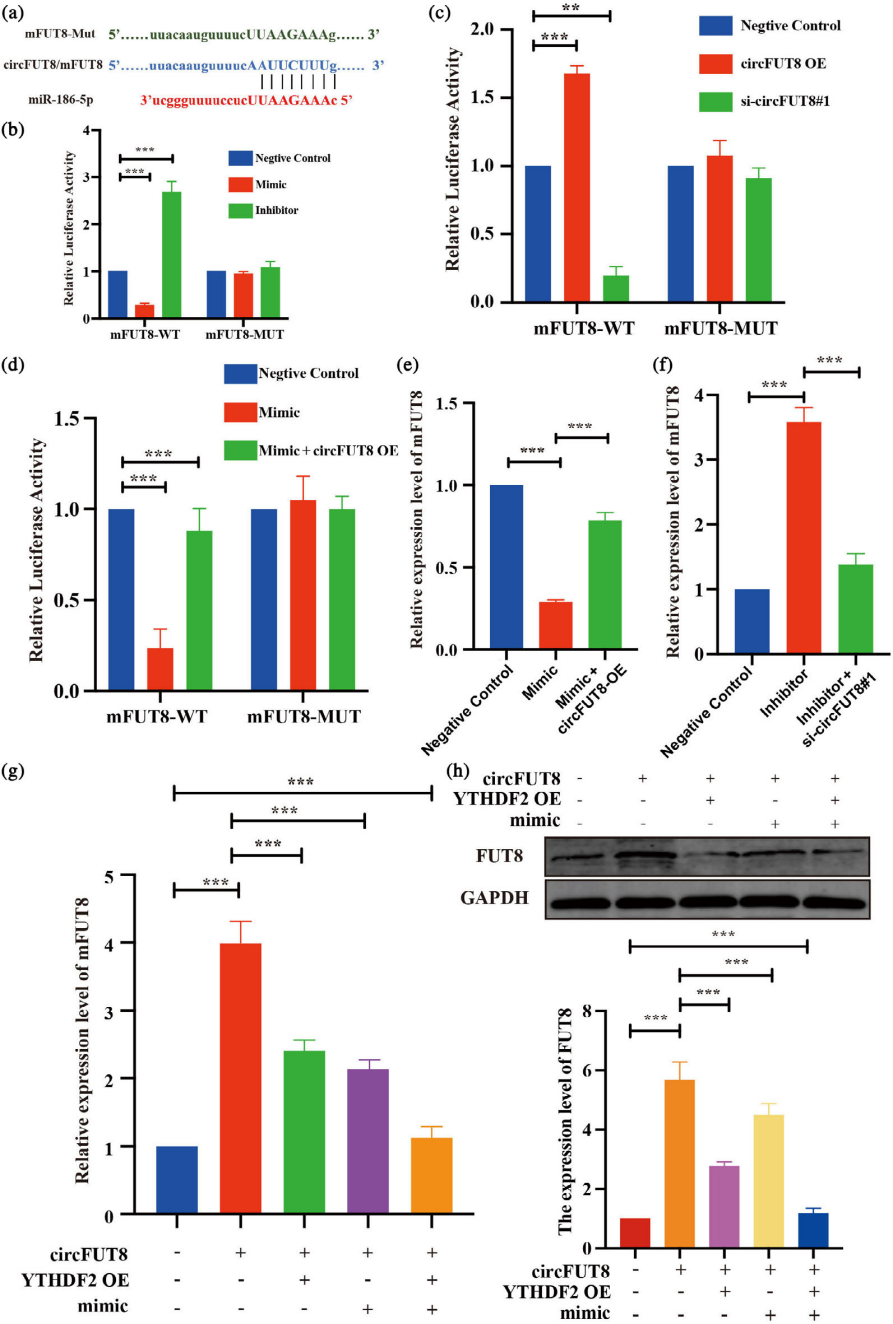
CircFUT8 could rescue both YTHDF2 and miR‐186‐5p induced the mFUT8 decrease. (a) A schematic drawing showing the putative binding sites of miR‐186‐5p to circFUT8 and mFUT8. (b) The luciferase activity of LUC‐mFUT8‐WT or LUC‐mFUT8‐MUT in A549 cells after transfection with negative control, miR‐186‐5p mimic or miR‐186‐5p inhibitor. (c) The luciferase activity of LUC‐mFUT8‐WT or LUC‐mFUT8‐MUT in A549 cells after transfection with negative control, circFUT8 overexpression plasmid, or circFUT8 siRNA. (d) The miR‐186‐5p mimic could significantly decrease the luciferase activity of LUC‐mFUT8‐WT, which was blocked by circFUT8. (e, f) The expression levels of circFUT8 could block the regulation ability of miR‐186‐5p in mFUT8. (g, h) Quantitative real‐time polymerase chain reaction (qRT‐PCR) and western blot showed that circFUT8 could promote the expression of mFUT8 and that the promotion could be blocked by overexpressed YTHDF2 and miR‐186‐5p.

Through TargetScan and miRanda analyses, we found that miR‐186‐5p bind to the region of mFUT8, which is also present in the sequence of circFUT8. According to the core region, we designed the wild‐type and mutation luciferase reporter plasmid of mFUT8 (mFUT8‐WT, mFUT8‐MUT) to verify the interaction (Figure 6a). The luciferase reporter assay showed that transfection of miR‐186‐5p mimic or inhibitor significantly influenced the luciferase activity of mFUT8, which could not affect the mFUT8‐MUT (Figure 6b). Similar results were observed when manipulating the expression of circFUT8 (Figure 6c). Subsequent rescue experiments showed that transfecting circFUT8 overexpression plasmid into miR‐186‐5p inhibitor cells significantly counteracted the miR‐186‐5p‐mediated suppression of mFUT8 expression (Figure 6d, e), and silencing of circFUT8 attenuated the mFUT8 expression induced by miR‐186‐5P inhibitor (Figure 6f). Additionally, overexpressing YTHDF2 and miR‐186‐5p simultaneously completely suppressed the increased expression of mFUT8 result from the overexpression of circFUT8, both at transcription and protein level, and knocking down YTHDF2 and miR‐186‐5p can also rescue the decrease in FUT8 expression caused by circFUT8 knockdown (Figure 6g, h and Figure S2F).

### CircFUT8 promote malignancy in LUAD dependent on mFUT8

**FIGURE 7:**
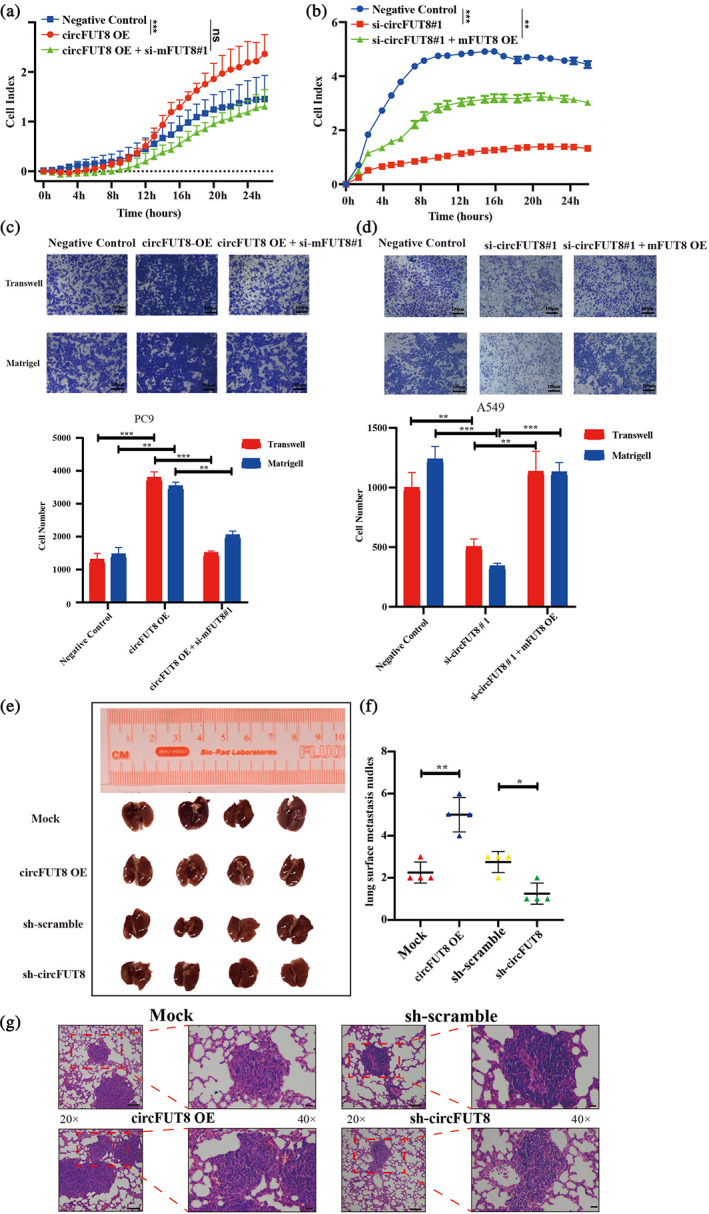
CircFUT8 promotes malignancy in lung adenocarcinoma (LUAD) dependent on mFUT8. (a) Real‐time cell analysis (RTCA) showing the invasion of PC9 cells stably transfected with negative control, circFUT8 overexpression plasmid or cotransfected with them and monitored by xCELLigence. (b) RTCA showing the invasion of A549 cells stably transfected with negative control, circFUT8 siRNA or cotransfected with them and monitored by xCELLigence. (c, d) Transwell and Matrigel assays indicated that mFUT8 could block the restrain invasion and migration, which were induced by circFUT8. (e, f) Representative images showing the decreased or increased tumor metastasis formed in the lungs of nude mice following vein tail injection of circFUT‐overexpression PC9 cells or circFUT8‐knockdown A549 cells. Metastases are indicated by arrows. (g) Representative hematoxylin and eosin (H&E) staining of lung metastatic lesions. 20×, scale bar, 20 μm. 40×, scale bar, 50 μm.

We have identified that circFUT8 interacted with YTHDF2 and miR‐186‐5p to stabilize the expression of mFUT8 in the aforementioned results. The upregulation of mFUT8 has been observed in several malignant cancers including liver, ovarian, thyroid, and colorectal cancers.^29^, ^30^ It has been reported that FUT8 globally regulates dozens of genes associated with malignant progression in NSCLC, including surface antigens, receptors, and adhesion molecules.^31^ We then explored whether circFUT8 function was dependent on mFUT8 in LUAD. The overexpression plasmid and siRNA of mFUT8 were efficiently constructed (Figure S2G, H). RTCA assay demonstrated that knockdown of mFUT8 functionally rescued the enhanced invasion upon circFUT8 overexpression, and the overexpression of mFUT8 also neutralized the metastasis upon circFUT8 knockdown (Figure 7a, b). Additionally, transwell and matrigel assays showed that circFTU8 elevated invasion and migration, which depended on the function of mFUT8 (Figure 7c, d). Moreover, we also explored the effect of circFUT8 in vivo through tail vein injection of circFUT8 overexpression, knockdown, and paired control cells, respectively, in BALB/c nude mice (Figure 7e). The results showed that overexpression of circFUT8 increased the metastasis and knockdown of circFUT8 inhibited the metastasis in LUAD (Figure 7f, g). Using IHC, we revealed that overexpression of circFUT8 led to upregulation of FUT8 protein in cancer tissues, while knockdown of circFUT8 showed the opposite effect (Figure S2I).

## DISCUSSION

**FIGURE 8:**
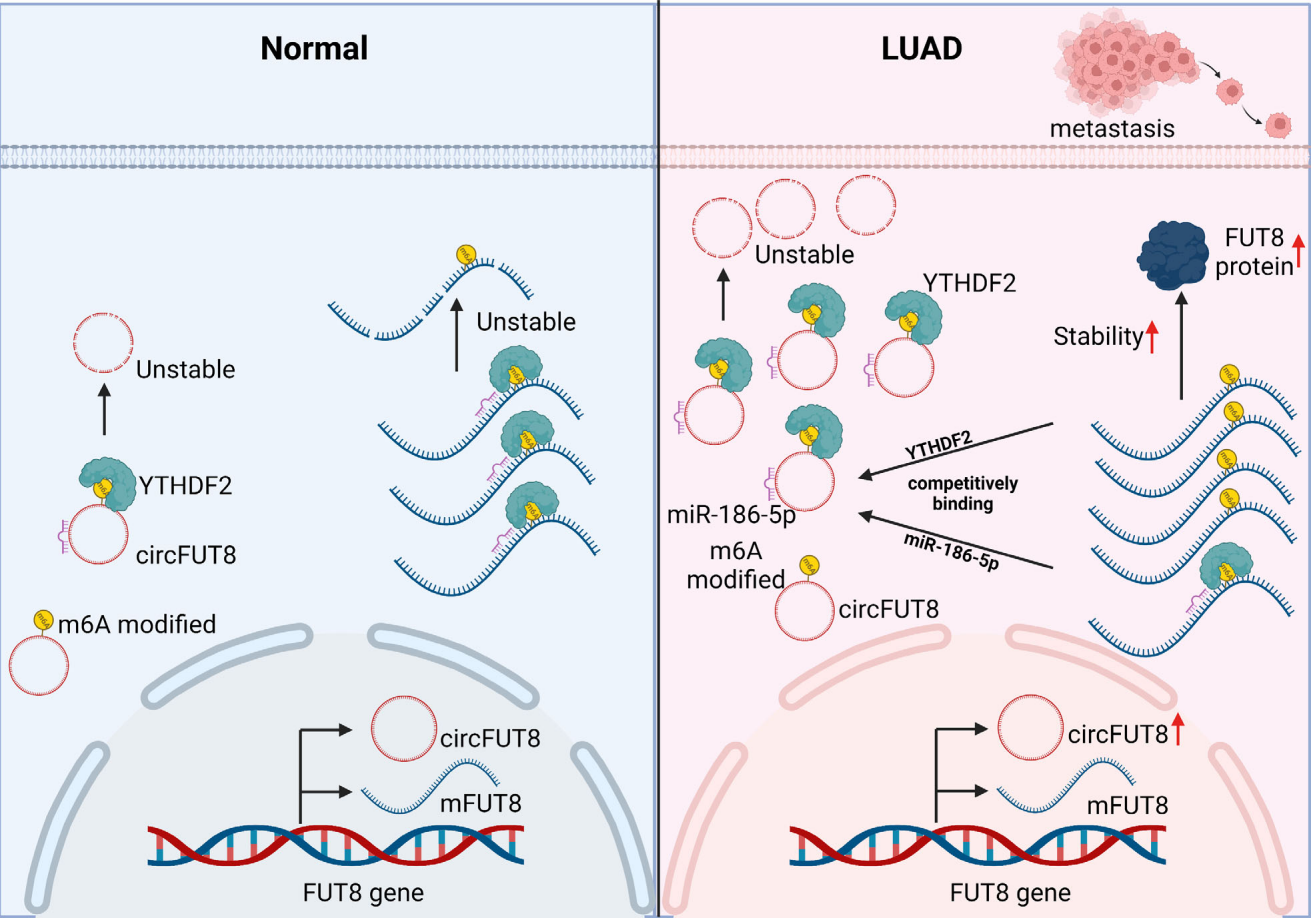
Schematic diagram showing the proposed mechanisms of circFUT8 in lung adenocarcinoma (LUAD).

In recent years, m6A modification of RNA, one of the most important ways of epitranscriptome,^32^ has been observed to be involved in the regulation of gene expression. In this study, we identified that circFUT8, derived from the exon 3 of the protein coding gene FUT8, was modified by the m6A methylation and recognized by the m6A “reader” protein YTHDF2. We also revealed that YTHDF2 not only interacted with circFUT8, but also combined with the line form mFUT8, inducing degradation both dependent on the HRSP12‐RNase P/MRP axis. CircFUT8 could competitively bind YTHDF2 to increase the stability of mFUT8 dependent on the m6A manner. Additionally, circFUT8 sponged miR‐186‐5p to increase the expression of mFUT8. Our study demonstrated that circFUT8 could interact with both YTHDF2 and miR‐186‐5p to function as a tumor oncogene dependent on the parent protein of FUT8 (Figure 8).

The crosstalk between m6A modification and circRNAs is attractive. Ye and colleagues developed a new bioinformatic tool called Circm6A to explore the transcriptome landscape of m6A modification in circRNAs.^33^ They found that m6A modified circRNAs tended to be hypermethylated in pancreatic ductal adenocarcinoma (PDAC) tumor species compared with adjacent normal tissues, which caused a gain of circRNA‐mRNA coexpression. In our study, we observed the positive correlation between circFUT8 and mFUT8. We revealed that both circFUT8 and mFUT8 interacted with m6A “reader” protein YTHDF2. Additionally, circFUT8 competitively combined with YTHDF2, which increased the stability of mFUT8.

The biological function of m6A modified circRNAs is partly determined by the m6A reader protein.^34^ It has been reported that the downregulated circNDUFB2 functions as a scaffold to enhance the interaction between TRIM25 and IGF2BPs, which mediated the degradation of IGF2BPs enhanced by m6A modification of circNDUFB2.^35^ The highly expressed circMAP3K4 encoded circMAP3K4‐455aa, which was driven by m6A modification, the m6A “reader” protein IGF2BP1, promoted hepatocellular carcinoma (HCC) progression by preventing HCC cells from cell death under stress.^36^ Additionally, the m6A modification also affected the stability of target circRNAs.^37^ The upregulated circRNA‐SORE was necessary for the maintenance of sorafenib resistance, which resulted from its increased RNA stability by the increased level of m6A at a specific adenosine in circRNASORE.^38^ In this study, we identified that YTHDF2 recognized circFUT8 to mediate its destabilization through HRSP12‐RNase P/MRP axis. CircRNAs are globally degraded by RNase L initiated by poly(I:C) stimulation or viral infection, which is required for PKR activation in early cellular innate immune response.^39^ However, the mechanism of circRNAs degradation has not been well elucidated, which is an interesting area for further research.

The correlation between the expression of circRNAs and their parent genes is also very intriguing.^40^ The landscape of circRNAs across many types of cancer revealed that the Spearman's rank correlations of most circRNAs and their parental expression were low.^41^ However, some studies revealed the regulatory relationship between circRNAs and the parental genes. Circ‐SNRK directly interacted with miR‐133 to increase the protein level of SNRK through preventing miR‐133 from directly binding to SNRK 3'UTR to suppress its translation,^42^ which proposed a positive relationship between circRNA and its parental gene. Interestingly, circPABPN1 interacted with RNA binding protein HuR to prevent HuR binding to PABPN1 mRNA and lowered PANPN1 translation,^43^ which revealed a negative correlation. In our study, we identified a positive relationship between the expression of circFUT8 and mFUT8. We also revealed that circFUT8 could sponge both YTHDF2 and miR‐186‐5p to increase the expression of mFUT8. Additionally, we also proved that circFUT8 functions as the tumor oncogene dependent on the parental coding protein FUT8. We hypothesized that due to the sequence similarity between circRNAs and their parental genes, the regulatory relationship was not solely limited to m6A modification or miRNA sponges, and also included RNA‐binding proteins and other manners of epitranscriptome.

In summary, our study revealed that circFUT8 increased the expression of its parental gene FUT8 both dependent on the m6A modification and miRNA sponge. Additionally, the oncogenic function of circFUT8 relies on the protein coding gene FUT8. Our study provides a novel aspect to explore the relationship between circRNAs and their parental genes.

## AUTHOR CONTRIBUTIONS

Yaping Wang and Feng Jiang designed the study. Data analysis, figure preparation, manuscript drafting and most of the experiments were performed by Gaochao Dong, Yingkuan Liang, and Bing Chen. Te Zhang, Hui Wang, Yuzhong Chen, and Yijian Zhang assisted with some of the experiments. All authors read and approved the final manuscript.

## CONFLICT OF INTEREST STATEMENT

The authors have declared no conflict of interest.

## Supporting information


**FIGURE S1.** Screening and characteristics of circFUT8 (A) The heatmap indicated the differential expressed profile of circRNAs in GSE101586, the red arrow annotated the circFUT8. (B) The expression levels of circFUT8 in the human bronchial epithelial (HBE) cell and LUAD cell lines. (C) PCR analysis for the expression of circFUT8 and mFUT8 after treatment with RNase R in the total RNA of H1975 cells. (D) The relative RNA levels of circFUT8 and mFUT8 were analyzed by qRT‐PCR after treatment with Actinomycin D at the indicated time points in H1975 cells. (E) Random hexamer or oligo(dT)18 primer used in reverse transcription experiments, and the analysis of the circFUT8 and mFUT8 levels by qRT‐PCR.

**FIGURE S2.** The efficiency of overexpression or knock‐down of YTHDF2, POP1, HRSP12, and mFUT8. (A) The qRT‐PCR and western blot validated the efficiency of YTHDF2 overexpression plasmid. (B) The qRT‐PCR and western blot were validated the efficiency of YTHDF2 siRNA. (C) The efficiency of POP1 and HRSP12 siRNA, respectively. (D, E) The western blot assay and DNA gel indicated that POP1 and HRSP12 could decrease the expression of circFUT8, mFUT8, and FUT8 protein. (F) qRT‐PCR showed that circFUT8 regulated the expression level of mFUT8 depending on both YTHDF2 and miR‐186‐5p. (G, H) The efficiency of overexpression and knockdown of mFUT8. (I) Representative images from immunohistochemical staining of FUT8 in tumor tissues from (G) scale bar, 80 μm.

**TABLE S1.** Primers, Probes, and RNA sequences used in this study.
**TABLE S2.** The antibodies used in this study.

## Data Availability

The accession number for the high‐throughput sequencing data reported in this article is NCBI GEO DataSets: GSE85324, GSE101586, and GSE49339. The data supporting the conclusions of this article have been provided in this article and its additional files. All other data from this study can be obtained from the corresponding author upon reasonable request.
