# Supplementary figures and images for: N 6‐methyladenosine‐modified circFUT8 competitively interacts with YTHDF2 and miR‐186‐5p to stabilize FUT8 mRNA to promote malignant progression in lung adenocarcinoma
Source: Thorac Cancer. 2023 Sep 5;14(29):2962–75. doi: 10.1111/1759-7714.15086 (PMC10569907; doi:10.1111/1759-7714.15086)

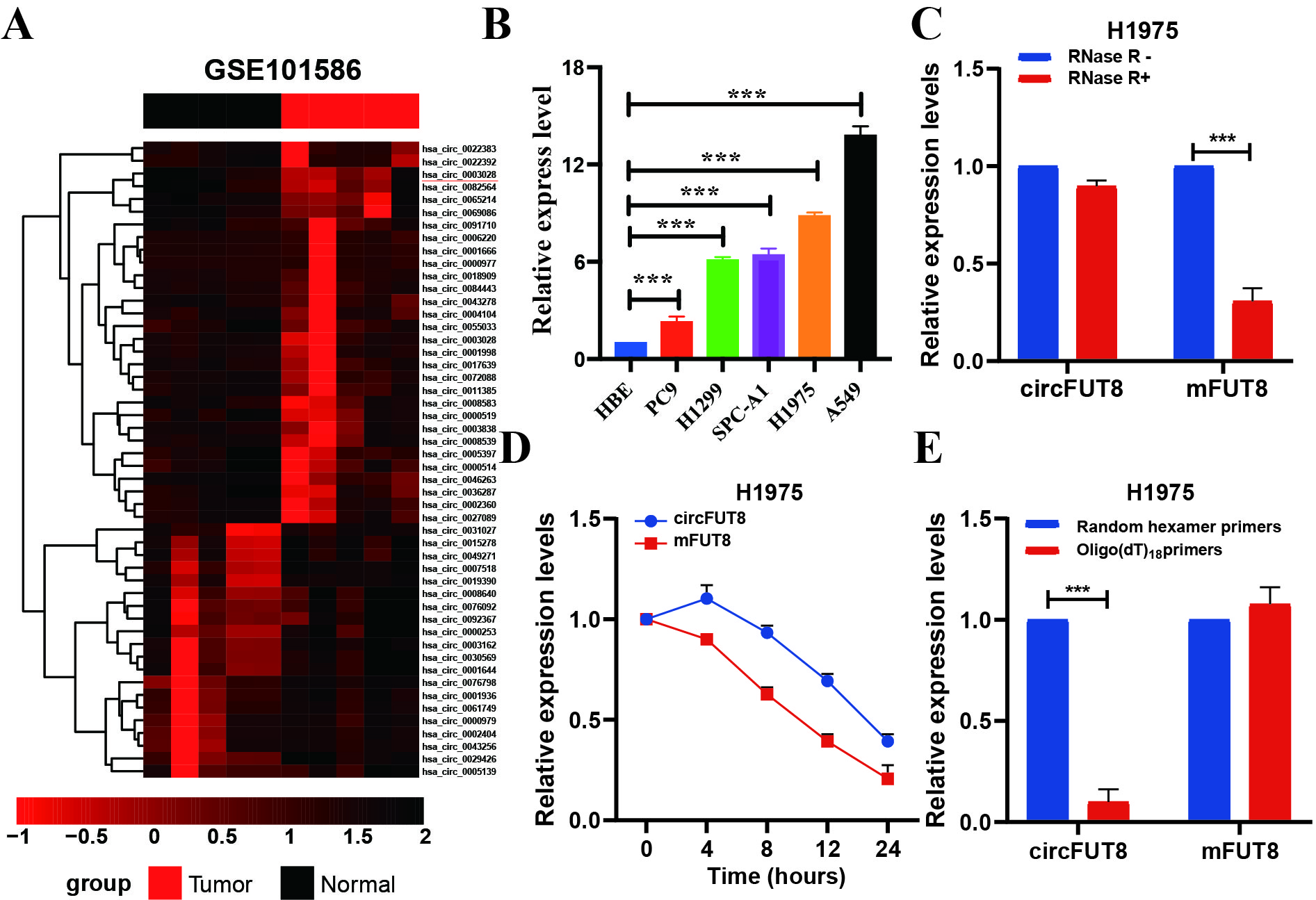

Supplement: Supplementary file 1 — FIGURE S1. Screening and characteristics of circFUT8 (A) The heatmap indicated the differential expressed profile of circRNAs in GSE101586, the red arrow annotated the circFUT8. (B) The expression levels of circFUT8 in the human bronchial epithelial (HBE) cell and LUAD cell lines. (C) PCR analysis for the expression of circFUT8 and mFUT8 after treatment with RNase R in the total RNA of H1975 cells. (D) The relative RNA levels of circFUT8 and mFUT8 were analyzed by qRT‐PCR after treatment with Actinomycin D at the indicated time points in H1975 cells. (E) Random hexamer or oligo(dT)18 primer used in reverse transcription experiments, and the analysis of the circFUT8 and mFUT8 levels by qRT‐PCR. [file TCA-14-2962-s003.jpg]

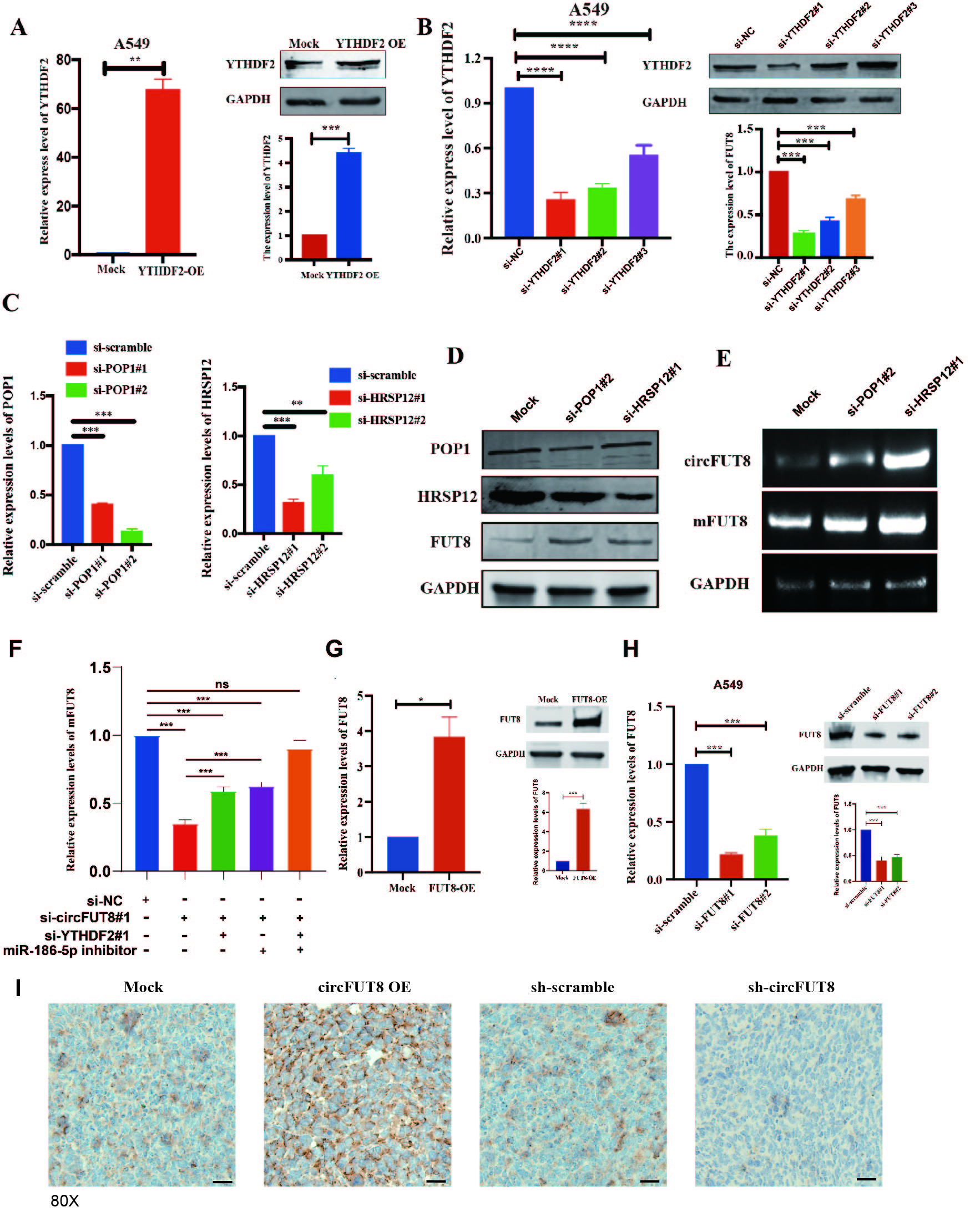

Supplement: Supplementary file 2 — FIGURE S2. The efficiency of overexpression or knock‐down of YTHDF2, POP1, HRSP12, and mFUT8. (A) The qRT‐PCR and western blot validated the efficiency of YTHDF2 overexpression plasmid. (B) The qRT‐PCR and western blot were validated the efficiency of YTHDF2 siRNA. (C) The efficiency of POP1 and HRSP12 siRNA, respectively. (D, E) The western blot assay and DNA gel indicated that POP1 and HRSP12 could decrease the expression of circFUT8, mFUT8, and FUT8 protein. (F) qRT‐PCR showed that circFUT8 regulated the expression level of mFUT8 depending on both YTHDF2 and miR‐186‐5p. (G, H) The efficiency of overexpression and knockdown of mFUT8. (I) Representative images from immunohistochemical staining of FUT8 in tumor tissues from (G) scale bar, 80 μm. [file TCA-14-2962-s002.jpg]
