# Supplementary material for: N 6‐methyladenosine‐modified circFUT8 competitively interacts with YTHDF2 and miR‐186‐5p to stabilize FUT8 mRNA to promote malignant progression in lung adenocarcinoma
Source: Thorac Cancer. 2023 Sep 5;14(29):2962–75. doi: 10.1111/1759-7714.15086 (PMC10569907; doi:10.1111/1759-7714.15086)
Supplement: Supplementary file 3 — TABLE S1. Primers, Probes, and RNA sequences used in this study. TABLE S2. The antibodies used in this study. [file TCA-14-2962-s001.docx]

**Table S1 Primers, Probes, and RNA sequences used in this study**

| **Primers for qRT-PCR** | | | |
| --- | --- | --- | --- |
| **Primer Name** | | **Sequence (5’-3’)** | |
| **circFUT8-F** | | **AGCCGAGAACTGTCCAAGAT** | |
| **circFUT8-R** | | **TCCTGTACTTCATGCGCTCT** | |
| **mFUT8-F** | | **AACTGGTTCAGCGGAGAATAAC** | |
| **mFUT8-R** | | **TGAGATTCCAAGATGAGTGTTCG** | |
| **Pre-FUT8-F** | | **TTGTACTTGGTGACTTGCGC** | |
| **Pre-FUT8-R** | | **TTTTGATCAGGGGCCCTTCT** | |
| **YTHDF2-F** | | **AGCCCCACTTCCTACCAGATG** | |
| **YTHDF2-R** | | **TGAGAACTGTTATTTCCCCATGC** | |
| **METTL3-F** | | **TTGTCTCCAACCTTCCGTAGT** | |
| **METTL3-R** | | **CCAGATCAGAGAGGTGGTGTAG** | |
| **METTL14-F** | | **AGTGCCGACAGCATTGGTG** | |
| **METTL14-R** | | **GGAGCAGAGGTATCATAGGAAGC** | |
| **WTAP-F** | | **CTTCCCAAGAAGGTTCGATTGA** | |
| **WTAP-R** | | **TCAGACTCTCTTAGGCCAGTTAC** | |
| **POP1-F** | | **AGAGGTGTAAAGCACCACAGT** | |
| **POP1-R** | | **GCTGTCGTGAAGTTCCAGG** | |
| **HRSP12-F** | | **GGGCCATTGGACCCTACAG** | |
| **HRSP12-R** | | **CGTTAGTGAAGTCACAGCCTG** | |
| **miR-186-5p-F** | | **AAGAATTCTCCTTTTGGGCT** | |
| **miR-186-5p-R** | | **GTGCGTGTCGTGGAGTCG** | |
| **GAPDH-F** | | **GGAGCGAGATCCCTCCAAAAT** | |
| **GAPDH-R** | | **GGCTGTTGTCATACTTCTCATGG** | |
| **U6-F** | | **CTCGCTTCGGCAGCACA** | |
| **U6-R** | | **AACGCTTCACGAATTTGCG** | |
| **Pull-down probe** | | | |
| **circFUT8** | | **Biotin-GAATCTCTCCGCATGTAGAGCGCA** | |
| **mFUT8** | | **Biotin-CTTCCCGTAGCCGTCCCTGGTCAA** | |
| **miR-186-5P** | | **Biotin-AGCCCAAAAGGAGAATTCTTTG** | |
| **FISH probe** | | |  |
| **circFUT8** | | **Cy3-GAATCTCTCCGCATGTAGAGCGCA** |  |
| **mFUT8** | | **Cy3-CTTCCCGTAGCCGTCCCTGGTCAA** |  |
| **miR-186-5P** | | **FITC-AGCCCAAAAGGAGAATTCTTTG** |  |
| **siRNAs** | |  |  |
| **Oligo set** | **Target sequences** |  |  |
| **si-circFUT8_1** | **CTCCGCATGTAGAGCGCAT** |  |  |
| **si-circFUT8_2** | **CCGAATCTCTCCGCATGTA** |  |  |
| **si-FUT8#1** | **GCCGAGAACTGTCCAAGATTT** |  |  |
| **si-FUT8#2** | **GCCAAGAAGCTAGTGTGTATT** |  |  |
| **si-YTHDF2_1** | **TACTGATTAAGTCAGGATTAA** |  |  |
| **si-YTHDF2_2** | **CGGTCCATTAATAACTATAAC** |  |  |
| **si-YTHDF2_3** | **GTGCATACAGTTTTCTA** |  |  |
| **si-POP1_1** | **GAATTTAACCGTAGACAAA** |  |  |
| **si-POP1_2** | **GATAGAAACCTGTAAGAAA** |  |  |
| **si-HRSP12_1** | **TGTAATAGGGAGAGTTGAA** |  |  |
| **si-HRSP12_2** | **TCTGCCTCCTGGGTTCAAG** |  |  |
| **si-scramble** | **GAAAAACAGGCACCTGGCTTG** |  |  |

**Table S2** The antibodies used in this study.

**Table S2 The antibodies used in this study**

| **Antibody** | **Supplier** | **Catalogue number** | **Host** |
| --- | --- | --- | --- |
| **FUT8** | **abcam** | **ab191571** | **R** |
| **YTHDF2** | **abcam** | **ab220163** | **R** |
| **POP1** | **proteintech** | **12029-1-AP** | **R** |
| **HRSP12** | **proteintech** | **12930-1-AP** | **R** |
| **N6-Methyladenosine (m6A)** | **CST** | **#56593** | **R** |
| **GAPDH** | **CST** | **#5174** | **R** |
